# Working on wellness (WOW): A worksite health promotion intervention programme

**DOI:** 10.1186/1471-2458-12-372

**Published:** 2012-05-24

**Authors:** Tracy L Kolbe-Alexander, Karin I Proper, Estelle V Lambert, Marieke F van Wier, Julian D Pillay, Craig Nossel, Leegale Adonis, Willem Van Mechelen

**Affiliations:** 1 UCT/MRC Research Unit for Exercise Science and Sports Medicine, Department of Human Biology, UCT School of Health Sciences, University of Cape Town, Cape Town, South Africa; 2 Department of Public and Occupational Health, and the EMGO Institute for Health and Care Research, VU University Medical Center, Amsterdam, The Netherlands; 3 Discovery Health, Johannesburg, South Africa; 4 MRC/UCT Research Unit for Exercise Science and Sports Medicine Department of Human Biology, Faculty of Health Sciences University of Cape Town, P.O. Box 115, Newlands, Cape Town, 7725, South Africa

## Abstract

**Background:**

Insufficient PA has been shown to cluster with other CVD risk factors including insufficient fruit and vegetable intake, overweight, increased serum cholesterol concentrations and elevated blood pressure. This paper describes the development of Working on Wellness (WOW), a worksite intervention program incorporating motivational interviewing by wellness specialists, targeting employees at risk. In addition, we describe the evaluation the effectiveness of the intervention among employees at increased risk for cardiovascular disease.

**Methods:**

The intervention mapping (IM) protocol was used in the planning and design of WOW. Focus group discussions and interviews with employees and managers identified the importance of addressing risk factors for CVD at the worksite. Based on the employees’ preference for individual counselling, and previous evidence of the effectiveness of this approach in the worksite setting, we decided to use motivational interviewing as part of the intervention strategy. Thus, as a cluster-randomised, controlled control trial, employees at increased risk for CVD (N = 928) will be assigned to a control or an intervention group, based on company random allocation. The sessions will include motivational interviewing techniques, comprised of two face-to-face and four telephonic sessions, with the primary aim to increase habitual levels of PA. Measures will take place at baseline, 6 and 12 months. Secondary outcomes include changes in nutritional habits, serum cholesterol and glucose concentrations, blood pressure and BMI. In addition, healthcare expenditure and absenteeism will be measured for the economic evaluation. Analysis of variance will be performed to determine whether there were significant changes in physical activity habits in the intervention and control groups at 6 and 12 months.

**Discussion:**

The formative work on which this intervention is based suggests that the strategy of targeting employees at increased risk for CVD is preferred. Importantly, this study extends the work of a previous, similar study, Health Under Construction, in a different setting. Finally, this study will allow an economic evaluation of the intervention that will be an important outcome for health care funders, who ultimately will be responsible for implementation of such an intervention.

**Trial registration:**

United States Clinical Trails Register NCT 01494207

## Background

Like many developing nations, South Africa has a dual burden of disease with non-communicable disease (NCD) accounting for more than a third (37%) of all death [1]. The other major causes of death are HIV/AIDS (30%) and other communicable diseases (21%) [1]. In addition to the increasing prevalence of NCD mortality and morbidity, there is a concomitant increase in the prevalence of contributing risk factors such as inactivity and obesity [2].

Indeed, South African employees are at increased risk for cardiovascular disease with more than half not meeting recommended physical activity guidelines [3]. Similarly, Prochaska et al., 2009 reported that 71% of the employees studied were inactive [4]. Furthermore, 80% of the employees were categorised as being at risk for 2 behavioural risk factors (which included inactivity, overweight or obese, smoking, not managing stress effectively) while 18% had three behavioral risks [4]. The workplace has been identified as a setting that can potentially reach a large number of people simultaneously, and positively impact on the risk and health profile of individuals [5,6], and is therefore an opportune setting for interventions targeting the adult population.

A health risk assessment (i.e. screening) has been regarded as an entry point in comprehensive health promotion programs and precedes the implementation of targeted interventions [7][8]. Those individuals identified as being at risk for cardiovascular disease may therefore be directed to appropriate intervention programs [9] aimed at reducing future healthcare expenditure. Interventions targeting those categorised as ‘high risk’ or ‘moderate risk’ [7] are likely to show greater improvements in health [9]. This is supported by Groeneveld et al., (2010) who conducted a recent review and reported that the health benefits from worksite interventions for employees who were not at risk for cardiovascular disease were minimal, compared to those employees identified as being at risk who showed greater improvements [10].

In addition to influencing health and lifestyle behavior, worksite interventions have been shown to play a role in increasing productivity and decreasing health-care costs [11]. Economic evaluations have shown employers the potential economic benefits associated with implementing workplace-based health intervention programs [12][11]. Indeed, employers may implement health intervention programs, specifically in order to reduce health related expenses and to increase productivity [13]. For example, the cost of employer subsidised health care increased 16% in the United States during 2003, motivating employers to find ways to reduce employee-related expenses [13].

The focus of recent workplace-based health research in South Africa has been predominantly centred on HIV/AIDS and hazardous occupational exposures [14-16]. Subsequently, there is a paucity of data on the effectiveness of intervention programs aimed at increasing physical activity and improving the CVD risk profile of the South African workforce. In addition, there is currently limited data on the economic benefits of worksite intervention programs which focus on health and lifestyle behaviour for South African companies.

Therefore the main aim of to the WOW study was to develop and evaluate a worksite health promotion program on improving physical activity behaviour and associated biological risk factors for cardiovascular disease among South African employees at increased risk for these diseases. Additionally, we will conduct an economic evaluation to determine the associated cost-effectiveness of the worksite intervention on both health and work-related outcomes in South Africa. It is envisaged that this research study will contribute to determining the effectiveness of worksite intervention programs on both health and work-related outcomes in South Africa.

This paper describes the development of Working on Wellness (WOW), a worksite intervention program and the proposed methodology to evaluate the effectiveness among employees at increased risk for cardiovascular disease.

## Methods/Design

### Development of the intervention

A systematic approach was used for the development of the intervention that was based on the intervention mapping (IM) process, previously described by Bartholomew et al. [17]. The IM protocol uses a stepwise approach, comprising of 6 steps, which ensures that an evidence-based approach is applied when developing an intervention. These six steps are; 1) needs assessment; 2) defining program objectives and developing matrices; 3) selection of theories and practical strategies for intervention; 4) design of the intervention program; 5) adoption and implementation plan and 6) evaluation plan [17].

The use of motivational interviewing in a worksite setting, comprised of six counselling sessions has previously been described [18] and the findings [19] are promising for the worksite setting. The research conducted by Groeneveld and colleagues [18] will form the basis of the methodological approach in this research study.

### Step one: Needs assessment

The aim of the needs assessment, which was comprised of a literature review and focus group discussions (FGD), was to examine the extent to which employers and employees would support the implementation of a health and lifestyle-based intervention in the workplace. In addition, employer and employee perceptions and expectations of such worksite health promotion programs were interrogated. Thus, this step of developing the intervention established whether South African employees would be interested in an intervention and secondly, to define objectives of the intervention program.

#### Literature review

A cross-sectional research study describing the health and cardiovascular disease risk profile of South African employees found that most of the employees (> 50%), presenting for the health risk appraisal, were not meeting the recommendation of 30 minutes of moderate to vigorous physical activity on at least 5 days of the week [3]. Furthermore, insufficient physical activity was clustered with the presence of additional risk factors among employees such as insufficient fruit and vegetable intake (80%), smoking (61%), overweight or obesity (31%), increased serum cholesterol concentration (19%) and elevated blood pressure (12%) [3]. These results suggest that a reduction in multiple risk factors could potentially be achieved by increasing habitual physical activity and subsequently decrease the risk of morbidity and mortality [12]. Furthermore, by decreasing the total number of risk factors, it may be possible to reduce absenteeism [20]. These findings underscore the importance of comprehensive worksite intervention programs, targeting of risk factors for CVD in South African adults. Interventions using such targeted messages for physical activity and nutrition may be modified on the basis of the individual’s self reported stage of change. A research study investigating the effectiveness of a tailored intervention program found that the employees who received tailored information in 8 sessions over a two-week period increased and maintained their habitual levels of physical activity for 12 months [21]. Employees in the control group were those who joined the company’s wellness centre and showed a decline in habitual physical activity over the same period [21]. Similarly, a worksite-based lifestyle intervention aimed at improving physical activity and nutrition habits, which incorporated tailored messaging based on the Trans Theoretical.

Model and consisting of 7 face-to face consultations, was effective in increasing energy expenditure, fitness level and reducing coronary artery disease risk factors [22]. A recent review of worksite interventions reported on the effectiveness of randomised, controlled trials for either group based education, individual counselling or supervised exercise interventions [10]. The authors concluded that interventions targeting employees at increased risk resulted in greater changes in body fat and body weight, compared to the inventions which targeted ‘mixed’ employees [10]. Mixed employee designs refer to those interventions which included employees, whether or not they were at risk for cardiovascular disease. Furthermore, interventions which were comprised of supervised exercise were the least effective in improving clinical outcomes. Conversely, individual-based counselling interventions resulted in marked decreases in peripheral body fat [10]. Similarly, a meta-analysis of worksite interventions targeting both dietary behaviour and physical activity, reported moderate quality of evidence for significant reductions in Body Mass Index [23]. Furthermore, significant improvements at 12 months were observed for the Framingham risk score and the prevalence of metabolic syndrome among the employees who received an intervention after completing a health risk assessment, compared to those in the control group [24]. The intervention in this worksite-based research study was multi-faceted and included distribution of pedometers, weekly healthy snack cart, on-site exercise and weight control programs and monthly newsletters [24].

#### Focus group discussions and interviews

The second part of the needs analysis involved employee-based focus group discussions (FGD) (n = 8) and interviews with Human Resource Managers from 11 companies. A letter of invitation was sent to the Human Resource Manager in a convenience sample of companies which were based in Cape Town. The aim of these FGD’s was to determine what the health priorities of both the employer as well as employee’s, the types of health promotion programs (if any) they may be interested in implementing or participating in at their work place. Ethics approval for the focus groups was obtained from the University of Cape Town’s Human Ethics Research Committee.

The Human Resource Managers of these companies were subsequently invited to participate in an interview. Employees from the same companies were invited to participate in FGD’s. The guiding questions in the interview aimed to establish whether their worksites currently had intervention programmes, the respondent’s perception of health, their main health priorities and the role of the worksite and the individual in improving health. All the sessions were audio-recorded and later transcribed, after which data were analysed using the thematic analytic approach [25].

Two thirds (66%) of the companies reported that they had health promotion programs at their worksite and these programs were predominantly focused on HIV/AIDS education, screening and counselling. However, when asked to rank the health priorities at the worksite, both the employees and Human Resource Managers reported that in addition to HIV/AIDS, reducing risk for cardiovascular disease, decreasing Body Mass Index and improving nutrition were important for worksite health and wellness. More importantly, the Human Resource managers reported that they felt their worksites were well-placed to influence habitual levels of physical activity, improve nutrition and reduce Body Mass Index. Both the managers and employees felt that despite wanting to improve health, they lacked the knowledge and skills to affect healthy lifestyle changes that would reduce their risk of cardiovascular disease. Employees preferred individual-based counselling and intervention programs to those that were group-based.

In summary, the needs assessment clearly underscored the potential benefits that an intervention program could have on employee health. Furthermore, both employees and managers identified the importance of addressing risk factors for cardiovascular disease at the worksite, and that individually-based interventions were preferred over group-based interventions. These findings suggest that interventions should be comprehensive and include more than one lifestyle behaviour, they should target employees at increased risk, and should be delivered using either group-based education or individual counselling.

### Step 2: Defining performance objectives

In this step of intervention mapping, we identified the intended changes in behaviour that were to become the target or focus of the intervention. We specified both the program and the respective performance objectives. The determinants, for both promoting and inhibiting the behavior are also described in this step. Based on the focus group study and the cross sectional evaluation of South African employees [3] the main program objectives were identified. These program objectives and their specific performance objectives, which were based in part, on the focus group discussions, are presented in Table 1. Most employees identified healthy nutrition and regular physical activity as the main components of a healthy lifestyle. However, they were unaware of the physical activity guideline to accumulate 30 minutes of moderate intensity physical activity on most days of the week. The main barrier reported for being sufficiently physically active and following a healthy diet was insufficient time. Employees also mentioned competing work and family demands, leaving them little discretionary time to find opportunities to be physically active or cook healthy meals. Some employees said that they preferred off-site physical activity intervention programs or those that took place during normal working hours. These employees stated that they wanted to go home to their families as soon as their working day was over and would not attend a program held after hours.

**Table 1:** Programme and performance objectives identified for the Working on Wellness Programme

**Programme objective**	**Performance objective**
Reduce the total number of risk factors for cardiovascular disease	1. Invite employees at increased risk for cardiovascular disease to participate in the intervention.
	2. Inform and interpret employees’ health risk assessment results.
	3. Identify the risk factors that require improvement.
	4. Employee and wellness counsellor set goals and strategies to improve health measures such as blood pressure, Body Mass Index.
Increase employees’ habitual levels of physical activity	5. Employees set physical activity goals. Do not specify in detail, eg by mentioning a wellness specialist. Because at this phase, you don’t this yet.
	6. Employees seek opportunities to be physically active both at work and at home.
	7. Employees identify personal barriers to physical activity, and provide solutions to overcome the barriers.
Increase fruit and vegetable intake.	8. Employees aim to accumulate at least 30 minutes of physical activity on at least 5 days of the week.
	8. Employees aim to increase daily fruit and vegetable intake to 5 servings or more per day.
	9. Employees list and choose a variety of fruit and vegetables that they might enjoy.
	10. Employees develop strategies and ideas with the wellness specialist to prepare their food/meals (and not to buy fast food/take out meals).

The work environment also inhibited physical activity, as employees spent most of their time sitting. One environmental barrier identified was seasonality, as employees interviewed indicated that they would not be prepared to exercise outdoors during the winter months, when the sun set earlier. Employees felt that they generally followed healthier diets during summer, when their intake of salads was higher, and there was a greater variety of fruit and vegetables.

Thus, the main objectives of the intervention are to increase habitual levels of moderate and vigorous intensity physical activity (to meet guideline of 150 minutes per week) and to improve dietary behaviour, specifically increased fruit and vegetable intake. The reduction in the totalnumber of risk factors will play a role in achieving the long-term goal of reduced risk and burden of cardiovascular disease.

### Step 3: Theory-based methods and practical strategy

Based on the employees’ desire for individual counselling, and previous evidence of the effectiveness of this approach in the worksite setting [19], we decided to incorporate motivational interviewing as the intervention strategy.

Most worksite interventions are typically comprised of either group education, supervised exercise or individual-based counselling with a large variation in frequency and duration of the interventions [10]. Supervised group exercise interventions appeared to be the least effective in improving health status [10]. However, a recent randomised controlled trial, which incorporated individually-based wellness counselling using motivational interviewing techniques, resulted in sustained reductions in body weight at 12 months among employees who were overweight at baseline [19].

Motivational interviewing has been defined as ‘a directive, client centred counselling style for eliciting behaviour change by helping clients to explore and resolve ambivalence’ [26] and has been applied in various settings [27]. Groeneveld et al. found that employees participating in a worksite-based motivational interviewing intervention reduced their body weight by an average of 0.9 kg after one year, compared to an increase in weight in the control group [19]. The main aim of their research was to determine the effectiveness of an individually-based counselling intervention in Dutch male construction workers at risk of cardiovascular disease [18]. This was a 12-month randomised control trial comprising of three face-to-face and four telephonic counselling sessions in which motivational interviewing techinques were employed [18].

Motivational interviewing is a technique that is collaborative and involves the respectful guiding of an individual (employee) towards health behavior choices, and avoids authoritarian judgment, and instructional approaches [26]. The counselling is focused and goal-directed where the client determines the reasons for change, and not the counsellor. The central principles which encompass motivational interviewing include; expressing empathy, supporting self-efficacy, rolling with resistance by avoiding debate or arguments and assisting the client to examine the discrepancies between their current and desired behaviours and goals [18][26]. Further, the Trans Theoretical Model (TTM) can be incorporated into a motivational interviewing session whereby the employee’s readiness to change can be determined so that materials and messages can be targeted to match an individual’s specific needs [22,28,29].

The TTM defines five different stages of change for health behaviors which are; a) pre- contemplation and describes those who do not intend changing or improving their behavior; b) contemplation and refers to those who are considering a behavior change; c) preparation, which suggests that individuals are getting ready for behavior change, d) action where the individual is actively engaging in the behavior change such as being more physically active and e) maintenance where the improved behavior is sustained and becomes habitual [30].

Interventions using targeted messages for physical activity and nutrition will therefore alter the message based on the individual’s self reported stage of change.

The efficacy of one-on-one counselling sessions based on the TTM stage of change has previously been was compared to receiving only written information on healthy lifestyle behavior in Dutch office-based workers [22]. The sedentary employees in both groups increased their levels of habitual physical activity from ‘not active’ to ‘active’. In another study, employees who received tailored information in 7 sessions over a six-month period, which incorporated the motivational interviewing approach, increased and maintained their habitual levels of physical activity for 12 months. Employees in the control group were those who joined the company’s wellness center and showed a decline in habitual physical activity over the same period (16).

Thus, motivational interviewing was selected as the main technique for the intervention, which would be comprised of one-on-one counselling, would incorporate determining the employee’s readiness to change, and is based largely on the previously described protocol by Groeneveld et al., 2008 [18]. This approach involves wellness counsellors, who are trained to communicate the risk of cardiovascular disease, in a manner that is easily understood by the employee. The employee, together with the wellness specialist, will then identify potential barriers and determinants of improving lifestyle and subsequently reducing their risk for cardiovascular disease. Together they will decide upon strategies and actions to reduce their risk of disease by increasing habitual levels of physical activity and improving dietary habits.

### Step 4: Design of the intervention

Based on the first three steps of intervention mapping, it was determined that the intervention would be focused on employees at increased risk for cardiovascular disease, and that the program would be implemented by way of invitation for one-on-one counselling sessions with a wellness specialist.

#### Motivational interviewing

In the FGD’s employees had reported that they enjoy the wellness and screening days offered by their health insurer and employer, and that they value the expertise of the healthcare professionals conducting the testing. Thus, the wellness counsellors, all of whom have had previous paramedical training (e.g. physiotherapist, exercise physiologist, dietician) will receive Motivational Interviewing training, in the form of a 3-day workshop, followed by 3 months of active training, from an accredited tertiary institution. The decision to give the wellness specialists three months of active training was based on the need to establish competency in motivational interviewing prior to initiating the intervention.

The intervention will consist six counselling sessions, whereby the first and last sessions will be face to face while the remaining four sessions will be telephonic. Employees in the FGD preferred face-to-face interactions, but due to time and logistical constraints, telephonic consultations will be included. Furthermore, one-on-one counselling sessions incorporating motivational interviewing principles, and which include telephonic sessions have been shown to be effective [19]. The aim of the first face-to-face session is to establish goals and strategies to improve health behaviour. The following four sessions will allow the counsellor to monitor progress towards the goal, set new goals or to adjust strategies to achieve a previously determined goal. The final session will comprise of a re-assessment of health and clinical outcomes, and for the employee to set additional goals or to discuss strategies to maintain healthy behaviours achieved.

### Step 5: Adoption and implementation plan

#### Pilot study

A pilot study was conducted in a convenient sample of employees for 12 weeks. The aim of the pilot was to determine the feasibility of the interventions and also to allow the counsellors to gain experience and proficiency in motivational interviewing techniques.

Eight wellness counsellors, based in three different South cities, recruited a convenience sample of both male and female employees (n = 225). The baseline participant characteristics are presented in Table 2. Despite the mean serum cholesterol and blood pressure being within normal ranges, more that 80% of the employees volunteering to participate had two or more risk factors for cardiovascular disease.

**Table 2:** Baseline characteristics of participants in the pilot study

	**n**	**Mean**	**± Standard Deviation**
Age (years)	225	40.0	10.8
Men (% of total sample)	121	51%	
Body Mass Index (kg/ht2)	225	31.0	5.5
Cholesterol (mmol/l)	228	4.7	0.9
Diastolic Blood Pressure (mm Hg)	229	84.1	11.6
Physical Activity (min/wk)	225	63.0	120.9

The analysis of covariance (ANCOVA) was performed to measure the effectiveness of the 12-week intervention. Participants were grouped into being either at risk or not at risk for each of the individual risk factors, and then the baseline and follow up measures were compared for each specific risk factor. Diastolic blood pressure decreased significantly over the 12 weeks period among those whose baseline result placed them in the at risk group (Diastolic blood pressure more than 90 mmHg) from 95.8 mmHg ± 9.8 to 90.1 mmHg ± 13.7 (p < 0.0001). Similarly, those with 2 or more risk factors at baseline decreased their number of risk factors from 3.5 ±0.8 to 3.0 ± 1.0 at follow up (p < 0.0001). In addition, there was a significant group and time effect for Body Mass Index, where those who were overweight or obese (BMI >25) at baseline experiencing a slight decline at 12 weeks (data not shown). This is an encouraging finding as 83% of the participants were either overweight or obese at baseline.

The intervention was well received by employees participating in the pilot study. They were encouraged that their health results obtained at their company’s wellness days could be improved upon. Most employees chose to increase levels of physical activity and improve various aspects of their diet such as increasing fruit and vegetable intake and decreasing daily fat consumption. Thus the improvements in clinical measures, and also the positive feedback from employees, supported the planned intervention.

### Step 6: Evaluation plan

#### Study design

The intervention as developed in the previous steps will be evaluated in a cluster-randomised controlled trial in South African worksites with a 12-month follow-up. The two intervention groups will receive 6 counselling sessions over either a 3 or 6-month period, described in Table 3. The control group will receive usual care. These participants will be unaware of the other two groups participating in the research study.

**Table 3:** Timeline and contact schedule for employees in the intervention groups

**Week**	**6-month intervention group**	**3 month intervention group**
0	Wellness day at company. (Baseline measurement)	Wellness day at company. (Baseline measurement)
1	Face-to-face contact	Face-to-face contact
	(set goals, action plan)	(set goals, action plan)
4	Telephone contact	Telephone contact
	(monitor progress)	(monitor progress)
8	Telephone contact	Telephone contact
	(monitor progress)	(monitor progress)
12	Telephone contact	Face to face (final contact)
	(monitor progress)	
16	Telephone contact (monitor progress)	
20	Telephone contact	
	(monitor progress)	
24	Face to face (final contact)	
	(set new goal, plan way forward)	
26	First follow up measurement	
52	Second follow up measurement	First follow up measurement Second follow up measurement

#### Setting

The study will be conducted in cooperation with a major national private health insurer, Discovery Health. Large companies (>300 employees) that are clients of this insurer, offering health and wellness days and that are based in three South African cities will be eligible to participate in this study.

#### Study population

Over the course of two months, all employees from the eligible companies will be invited to participate in the wellness day(s) of their company. The health insurer will offer and conduct a Health Risk Assessment of risk factors, including: family history, Body Mass Index (BMI) and body fatness, blood pressure and finger-prick cholesterol and glucose concentrations, self-report physical activity and dietary intake behaviour, smoking, and stress, to the employees at the wellness day.

Those employees who are identified to be at ‘increased’ or ‘high’ risk for CVD, based on an adapted Framingham score of 10% or more [18] will be invited by the wellness specialists (via telephone call) to take part in the research study. Employees agreeing to participate will be asked to sign an informed consent and to provide their email address and contact details.

Because all the companies hosting wellness days in this research study are clients of a major national health insurer, it will be able to access data for employees who do not volunteer for the screening activities, and thus be able to compare responders to non-responders. Recorded data for all employees include healthcare expenditure and whether they participated in a health screening activity recently. The data for the employees who choose not to participate will be sent to the researchers without any personal identifiers, thus ensuring employee confidentially. Being able to describe the non-responders is one of the strengths of this study design, and the insured population with whom we are working, providing insight into the problem of selection bias.

#### Randomisation

Randomisation will take place at the company level, where participating companies will be assigned to either the intervention or the control group. The wellness specialists’ manager will assign each company with a number and then use a random numbers table to allocate the companies to either the intervention or control.

#### Blinding

Due to the nature of the research, the wellness specialists will not blinded and knew which companies were receiving the intervention. However, the employees participating in the research study were not aware of the expected outcomes of the study, nor were they aware of the other (either control or intervention) group participating in the research study.

#### Inclusion and exclusion criteria for companies

Companies with 300 or more employees will only be selected from those willing to allow a repeat of the HRA onsite at 6 and 12 months follow-up. In addition, the employer should have employee absenteeism data from their employee records available.

One of the challenges with worksite health promotion programs is that the company management should “buy in” to the intervention and support its implementation, and also “buy in” to the study and be willing to being placed in the wait-list control arm of the study. These issues will be discussed with the company during recruitment. To emphasize the importance of continued support, we require a letter of commitment from the management of prospective companies for the duration of the study. Furthermore, the researchers will invite participation and seek approval from the respective unions and health and safety committees functioning at the companies.

#### Inclusion and exclusion criteria for employees

Employees identified to be at ‘increased’ or ‘high’ risk for cardiovascular disease are eligible to take part in the research study. Risk status will be determined by using an adapted Framingham Score [18] together with habitual physical activity levels, and Body Mass Index. This score incorporates age, total cholesterol, blood pressure and smoking status and ranks individuals according to their level of risk, ranging from very low to very high 10-year risk of fatal CVD . Those members presenting for health risk appraisal with a risk of 10% or higher will be considered eligible. Independent of risk status, those employees who are obese (Body Mass Index > 30 kg/m2) also will be eligible to take part in the study. Only those employees who are include18 years or older and who have a contract with employer until end of 12-month measurement period will be invited to participate.

Employees will be excluded for the following reasons: pregnancy, diagnosis or treatment of cancer, any other disorder that makes physical activity impossible. Contract workers whose employ with the company will end before the 12 month follow up measurement, will also be excluded from the study.

#### Sample size calculation

**Figure 1:**
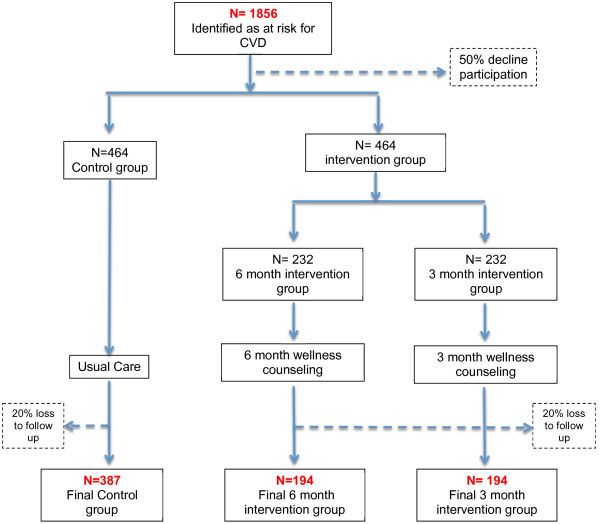
Flow diagram of participant recruitment.

The sample size was determined according to a power calculation using a dichotomous variable: meeting the PA guideline with at baseline 50% not meeting the guideline and after the intervention 40% (intervention group) vs 50% (in control group). Based on these numbers, and 80% power and significance of 0.05, 775 employees are required at the 12-month follow-up with 387 in control group and 387 in intervention group. Thus, assuming that approximately 20% of participants will be lost to follow-up at 12 months, we will aim to recruit 928 employees at baseline and allocate 424 to the intervention and control groups (Figure 1).

### Intervention group

The wellness specialists will calculate the adapted Framingham score for all employees who have voluntarily participated in their worksite’s wellness day. All employees who are identified as being at increased risk will receive a telephone call from the wellness specialist inviting them to participate in the research study. Should they agree, the wellness specialist will book the first face-to-face session, which will take place at the employee’s worksite.

Individuals in the intervention group will receive the same advice and health promotion materials as those in the control group. Additionally, they will be offered an individually tailored intervention that consists of one-on-one counselling sessions. The first intervention group will receive the six one-on-one counselling sessions over a 6-month period (6-I), while the second intervention group will receive the counselling over a 3-month period (3-I).

### Control group

The employees in the control group will receive usual care. This is comprised of feedback at the health and wellness day, directing employees to their physician if needed, and encouragingthem to increase their level of physical activity and to improve nutrition in a generic way. They will receive general health promotion material detailing the risks for cardiovascular disease and once-off advice on improving lifestyle behaviour. The participants from the control companies will also participate in follow up measurements at 6 and 12 months.

If the intervention is shown to be effective, the companies who were in the control group will receive the intervention after completion of the research study. Therefore, the control group will be wait-listed for the intervention.

### Outcome measures

The primary outcome of this study is a change in level of habitual daily physical activity. Secondary outcomes include: changes in dietary intake and Body Mass Index. Further, the secondary outcome measures include changes in the cardiovascular disease risk profile, which will be defined by the adapted Framingham score, incorporating blood pressure, total blood cholesterol, and smoking status. Data will be collected at baseline and at 6 and 12 months.

### Health risk appraisal (HRA) questionnaire

The HRA is comprised of demographic, health and lifestyle factors, as well as questions related to stages of change for the various risk behaviours. The demographic variables include age and gender; while the lifestyle measures include, fruit and vegetable intake, habitual alcohol consumption and habitual physical activity. For smoking, fruit and vegetable intake and habitual physical activity, the participant also reports on readiness to change or improve these behaviours. The questions on readiness to change are based on the Transtheoretical Model stages of change [6]. Additionally, the health screening will be comprised of the finger-prick capillary blood samples for serum cholesterol and glucose concentrations, blood pressure, height and weight measurements. All screening will conducted by qualified, trained staff, provided by the health insurer and will form part of their wellness day which is offered to their corporate clients.

### Habitual physical activity

Habitual physical activity will be measured using the ‘Global Physical Activity Questionnaire’ (GPAQ) [31]. This is a self administered questionnaire that includes questions on habitual levels of light, moderate and vigorous activity. The physical activity domains include work-related activity, transport and leisure time activity, which are then summed to calculate total minutes of physical activity per week. Participants will be categorised according to whether they meet the American College of Sports Medicine guideline of 150 minutes of physical activity per week.

#### Dietary habits

Habitual dietary fat intake will be measured using the Short Fat Questionnaire [32]. The data obtained from this questionnaire will supplement the information on habitual fruit and vegetable intake and general dietary habits obtained from the Health Risk appraisal.

#### Presenteeism and absenteeism

The Healthy Days Questionnaire devised and tested by the US Centers for Disease Control and Prevention, will be used to measure health-related quality of life. Healthy days are calculated using a series of 4 questions, focusing on general perceived health, self-rated physical and mental health and the extent to which physical and/or mental health may have limited work-related activity within the past 30 days [33]. Absenteeism data will be obtained from the company’s Human Resource office, in addition to the participant keeping a log book so that the wellness specialist can record days away from work at each consultation and at the 6 and 12 month follow up visits.

#### Clinical measures

The following clinical measures will be performed by the trained health professionals employed by the health insurer at the wellness days. Cholesterol screening will be conducted using finger-prick capillary blood samples (Accutrend ® GC analysers, Roche Diagnostics) to measure total serum cholesterol concentrations. Systolic and Blood pressure will be measured twice per person using an automated sphygmomanometer. Employees will be instructed to sit quietly for approximately three minutes before being measured. Both readings will be recorded and the average of the two will be used in the statistical analysis.

Standing height (cm) will be measured to the nearest 0.1 cm, using a stadiometer. Body weight will be measured using a portable calibrated scale and recorded to the nearest 0.1 kg. Employees will be asked to remove shoes, jackets/jumpers/jerseys and asked to empty pockets for these measurements. Body Mass Index (BMI) will be calculated as body mass (kg) divided by height (m) squared (kg/m2).

### Process measures

A process evaluation will be conducted to determine if the intervention was implemented as intended. The counsellors and participants will be asked to critically appraise the various components of the intervention and to grade their experience. This appraisal will include a selfreport on the frequency and ease of use of the key concepts and tools applied during the motivation interviewing session, such as the importance and confidence rulers. Counsellors will also record the number of telephone calls and emails sent prior to confirming an appointment with the employee, the number of missed and re-scheduled appointments and additional information that may be requested by the employee.

A sub-sample of participants will be contacted post the intervention and invited to a focus group discussion to relay their experiences and assessment of the counselling sessions. Fidelity and adherence to the motivational interviewing technique will be measured by the principle researcher, using the ‘Motivational Interviewing Treatment Integrity (MITI) scoring methodology which incorporates the random recording and evaluation of their audio recorded counselling sessions.

#### Costs

The intervention costs that will be included are the cost of the wellness days screening, training of counsellors, employment costs of counsellors, opportunity cost that may be lost if employee’s sessions are during working hours, health promotion materials, travel and telephone calls. These data will be obtained from the health insurer who will keep records of all expenses related to the intervention, which includes the duration of counselling sessions.

Direct health care costs will be based on health-related claims data linked to doctors visits, hospitalisation and chronic medication will be obtained from the medical insurer. Indirect costs include costs for loss of productivity (i.e. sickness absenteeism). Absenteeism data will be obtained from the company Human Resource manager in each company. In addition, participants will keep a medical costs and absenteeism diary and submit it to the research team every 3 months.

### Statistical analyses

STATISTICA software package will be used for all the analyses (Stasoft, Inc. 184–199, Tulsa OK, SA) and intention to treat analysis will be performed for each of the outcomes. Mean, standard deviation and standard error will be calculated for the continuous variables. Frequency tables will be used to determine the percentage of individuals at risk. An Analysis of Variance (ANOVA) will be performed to determine whether there were significant changes in physical activity habits and nutrition in the intervention and control groups at 6 and 12 months.

The cost-effectiveness of the intervention programme will be determined from the societal and the employer perspective. Confidence intervals around differences in costs between the intervention groups and control group will be estimated using bootstrapping techniques. Cost effectiveness ratios will be calculated by dividing the difference between the mean total costs of the intervention groups and control group, by the difference in the mean habitual physical activity levels and total number of risk factors for cardiovascular disease. Uncertainty around the cost-effectiveness ratios will be graphically presented on Cost-Effectiveness planes (CE-planes) [34]. Furthermore, Cost-effectiveness acceptability curves will be estimated to show the probability of cost-effectiveness at different ceiling ratios [35].

## Ethical considerations

Each employee participating in the wellness days and who is classified as being at increased risk for CVD, will receive an information sheet containing information on the purpose of the research and contact details of the principal investigators, and will be invited to participate in the intervention. Employees that agree to take part will be asked to sign a consent form prior to participating in the study. Participants will be assured that their participation in the research is voluntary, that they may withdraw at any time and that their withdrawal will not have a negative impact on their employment, and that they will continue to receive all usual care health insurance benefits and programmes. The participants will also be assured that their employer will not have access to any of the information collected for the research study, and that all information is confidential. The health insurer will send encoded data of employees declining to participate, in order to describe their health status and healthcare expenditure. There will be no personal identifiers, thus all information will be anonymous. This study will be conducted in accordance with the Declaration of Helsinki, Good Clinical Practice as well as the laws of the Republic of South Africa. The University of Cape Town’s Research Ethics Committee has given ethical approval for this research study (reference number: 044/2009).

## Discussion

We have identified a need for worksite health promotion programs in South Africa to address the increasing prevalence of CVD and the associated risk factors, which is the underlying rationale for the Working on Wellness - WOW - worksite intervention research study. This intervention aims to identify employees at increased risk for cardiovascular disease and provide them with counselling, through motivational interviewing, to equip them to reduce their risk factor profiles.

This approach is supported by previous research which showed that face to face counselling in the worksite resulted in increased physical activity, improved cardiovascular fitness, decreased body fat percentage and serum cholesterol concentrations [22]. In addition, both employers and employees participating in the formative assessment expressed a desire for interventions which address CVD and the associated risk factors. Furthermore, the preliminary results from the pilot study supports the high risk approach based on the trends in reduction in risk after only 12 weeks of intervention. Despite the promising results of the pilot study, the limitations of not having a control group needs to be acknowledged.

The adherence to the Motivational Interview techniques will be monitored during the course of the intervention where at least 12 audio-recordings per wellness specialist will be coded using the MITI scoring. Thus, this research study will also be able to provide more insight into the fidelity of the intervention.

Worksites have been identified as opportune settings for health promotion and intervention programs. However, there is still no conclusive evidence on the effectiveness of these programs on employee health, as reviews have shown that results are still ‘somewhat equivocal’ [36]. A need for further research in the worksite has been identified which includes randomised control trials in order to provide more evidence on the effectiveness of intervention programs [37]. This research study aims to contribute to the current knowledge and provide new insight into the ffectiveness of worksite intervention programs targeting employees at increased risk for cardiovascular disease. The economic evaluation of the intervention may also provide new knowledge on the cost effectiveness of one-on-one counselling for employees.

## Competing interests

TKA, KIP, EVL, MFvW, JDP and WvM declare that they have no competing interests. CN and LA are employed by a national health insurer, Discovery Health, and eight of their employees will be seconded to delivering the intervention in the worksites. There are no financial competing interests. There are no restrictions by the health insurer on publications. The research study was funded by a grant from the South Africa Netherlands Research Programmes on Alternatives in Development (SANPAD), and there are no competing interests between any of the authors and SANPAD.

## Authors’ contributions

TKA, KIP, EVL, MFvW, WvM, JDP, CN and LA: part of conception and design of intervention TKA, conducted focus group discussions, drafted the manuscript KIP, EVL, MFvW, JDP, WvM, CN: editing and revision of manuscript. All authors read and approved the final manuscript.

## Pre-publication history

The pre-publication history for this paper can be accessed here:

http://www.biomedcentral.com/1471-2458/12/372/prepub
